# Comparison of preventive effects of combined furosemide and mannitol versus single diuretics, furosemide or mannitol, on cisplatin-induced nephrotoxicity

**DOI:** 10.1038/s41598-024-61245-6

**Published:** 2024-05-07

**Authors:** Ayaka Takagi, Takanori Miyoshi, Toshinobu Hayashi, Hinako Koizumi, Kyouichi Tsumagari, Chiaki Yokota, Takafumi Nakano, Koichi Matsuo, Takashi Egawa

**Affiliations:** 1 Department of Emergency and Disaster Medical Pharmacy, Faculty of Pharmaceutical Sciences, Fukuoka University, 8-19-1, Nanakuma, Jonan-ku, Fukuoka, 814-0180 Japan; 2 Department of Pharmacy, National Hospital Organization Kyushu Medical Center, 1-8-1, Jigyohama, Chuo-ku, Fukuoka, 810-8563 Japan; 3 Department of Pharmacy, National Hospital Organization Beppu Medical Center, 1473 Ooazauchikamado, Beppu, 874-0011 Japan; 4 Department of Pharmacy, National Hospital Organization Okinawa Hospital, 3-20-14, Ganeko, Ginowan, Okinawa 901-22143 Japan; 5 Department of Pharmacy, National Hospital Organization Nagasaki Kawatana Medical Center, 2005-1, Shimogumigou, Kawatana-cho, Higashisonogi-gun, Nagasaki, 859-3615 Japan; 6 Department of Oncology and Infectious Disease Pharmacy, Faculty of Pharmaceutical Sciences, Fukuoka University, 8-19-1, Nanakuma, Jonan-ku, Fukuoka, 814-0180 Japan

**Keywords:** Cisplatin, Nephrotoxicity, Renal failure, Diuretics, Furosemide, Mannitol, Chemotherapy, Lung cancer

## Abstract

Cisplatin (CDDP)-induced nephrotoxicity is a common dose-limiting toxicity, and diuretics are often administered to prevent nephrotoxicity. However, the efficacy and optimal administration of diuretics in preventing CDDP-induced nephrotoxicity remain to be established. This study aimed to evaluate the efficacy of combining furosemide and mannitol to prevent CDDP-induced nephrotoxicity. This was a post-hoc analysis of pooled data from a multicenter, retrospective, observational study, including 396 patients who received one or two diuretics for CDDP-based chemotherapy, compared using propensity score matching. Multivariate logistic regression analyses were used to identify risk factors for nephrotoxicity. There was no significant difference in the incidence of nephrotoxicity between the two groups (22.2% vs. 28.3%, P = 0.416). Hypertension, CDDP dose ≥ 75 mg/m^2^, and no magnesium supplementation were identified as risk factors for nephrotoxicity, whereas the use of diuretics was not found to be a risk factor. The combination of furosemide and mannitol showed no advantage over a single diuretic in preventing CDDP-induced nephrotoxicity. The renal function of patients receiving CDDP-based chemotherapy (≥ 75 mg/m^2^) and that of those with hypertension should be carefully monitored. Magnesium supplementation is important for these patients.

## Introduction

Cisplatin (CDDP) is a platinum-containing drug and effective anticancer agent for a wide range of solid tumors that inhibits DNA replication by forming DNA crosslinks. CDDP has been reported to cause several side effects, including nephrotoxicity, myelosuppression, alopecia, deafness, nausea, and vomiting, of which nephrotoxicity is the most severe because renal failure significantly affects future drug treatments and subsequently results in the discontinuation of CDDP-based chemotherapy.

CDDP-induced nephrotoxicity is considered to be directly caused by the toxic effect of CDDP accumulation in tubular epithelial cells and secondary to CDDP-induced inflammation that increases the generation of reactive oxygen species and inflammatory mediators leading to tubular epithelial cell necrosis and apoptosis, resulting in hypomagnesemia and reduced renal function^1–5^.

Traditionally, intensive intravenous high-volume hydration before and after CDDP administration has been the mainstay of nephrotoxicity prevention^3^. Hydration reduces nephrotoxicity by increasing urine output, thereby reducing CDDP concentration in the kidneys. In recent years, a short-term low-volume hydration method has been developed and used in routine clinical practice, along with conventional high-volume hydration^6,7^.

Additionally, hypomagnesemia induces the saturation of active transport mechanisms in renal tubular cells, leading to excessive CDDP levels in renal tubular cells and subsequent cell necrosis. Therefore, magnesium (Mg) supplementation has been used to prevent nephrotoxicity, and its preventive effects have been described in several studies^7–11^.

Although controversial, diuretics have been reported to limit CDDP-induced nephrotoxicity. Diuretics decrease urinary CDDP concentrations by increasing water excretion and blocking chloride reabsorption, thereby decreasing the rate of CDDP activation by aquation^5,12,13^.

Commonly used diuretics in clinical practice include the osmotic diuretic mannitol and loop diuretic furosemide. Several studies have reported the protective effects of mannitol^4,14–17^. However, mannitol may contribute to hypomagnesemia by increasing Mg excretion^18^, and there is insufficient evidence to support the use of mannitol in forced diuresis.

Studies evaluating the role of furosemide in reducing CDDP-induced nephrotoxicity have reported conflicting results. Increased nephrotoxicity has been reported in rodents treated with furosemide^19^. Another in vivo study demonstrated the protective effect of reduced urinary platinum levels after furosemide administration prior to CDDP administration in rats^20^. Santoso et al. reported that hydration with saline or saline plus furosemide was associated with reduced CDDP-induced nephrotoxicity^21^. Although there is some consensus regarding the use of diuretics to prevent nephrotoxicity, the evidence is insufficient as there are many unknown aspects regarding the effects of diuretics. Furthermore, the efficacy and optimal administration of diuretics to prevent CDDP-induced nephrotoxicity are yet to be established.

Furosemide and mannitol are currently used in clinical practice, and the two can be used in combination. A previous study reported that approximately 30% of the patients undergoing CDDP-based chemotherapy received a combination of two diuretics for forced diuresis^22^. Whether the administration of dual diuretics is more effective than that of a single diuretic in preventing nephrotoxicity remains unclear. Therefore, this study aimed to investigate the efficacy of combining furosemide and mannitol in preventing CDDP-induced nephrotoxicity.

## Materials and methods

### Setting and patients

This study was a post-hoc analysis of pooled data from a multicenter, retrospective observational study conducted in five hospitals affiliated with the National Hospital Organization in Kyushu, Japan^22^. All participants were treated in accordance with the principles outlined in the Declaration of Helsinki. The Ethics Committee of Beppu Medical Center waived the requirement for informed consent owing to the retrospective nature of the study. Patient data were used after allowing patients to refuse to participate using an opt-out form. In this study, we analyzed the pooled data of 657 patients with cancer with Eastern Cooperative Oncology Group (ECOG) performance status (PS) of 0 to 2, creatinine clearance (CCr) ≥ 60 mL/min, and no history of CDDP administration and had received 20 mg of furosemide and/or 300 mL of 20% mannitol as forced diuresis with conventional high-volume hydration for each chemotherapy cycle. Furosemide and mannitol were given sequentially rather than concurrently when administering the two diuretics. In this study, patients with a short hydration method were excluded to ensure comparable hydration conditions for evaluating the effects of diuretics. Information on the cancer types and chemotherapy regimens of eligible patients is presented in Supplementary Tables 1 and 2.

### Data collection

Data on the following patient characteristics were collected: Mg dose, sex, age, primary cancer site, cancer stage, ECOG PS, presence of cardiac disease, presence of diabetes, presence of hypertension, chemotherapy regimen, CDDP dose, presence of short hydration, regular use of nonsteroidal anti-inflammatory drugs (NSAIDs), diuretic type, number of chemotherapy courses administered, serum creatinine (SCr) level and changes therein, CCr and changes therein, occurrence of renal failure, and Common Terminology Criteria for Adverse Events (CTCAE) ver. 5.0 grade. Cardiac disease was defined as angina pectoris, myocardial infarction, atrial fibrillation, arrhythmia, or valvular disease. SCr was measured using an enzymatic method at least 2 weeks after the start of CDDP administration and was used to determine the presence of renal impairment. CCr was calculated using the Cockcroft–Gault formula. The CTCAE^6–8,10,11,14,16^ and the Cockcroft–Gault formula^6–8,10,11^ are widely used for the assessment of renal function in the setting of cancer chemotherapy. Based on the CTCAE ver. 5.0 grades for creatinine increase, the development of renal impairment was defined as an increase in the SCr after CDDP administration of at least one grade higher than that before CDDP administration. All patients received conventional high-volume hydration, and none of them received short hydration.

### Statistical analysis

Patient characteristics and incidence of nephrotoxicity were summarized using descriptive statistics or contingency tables and were compared using the Mann–Whitney U test and Chi-square test. Propensity score matching was used to reduce bias and balance patient characteristics between the one- and two-diuretic groups. A propensity score calculated using logistic regression analysis was used for this purpose (covariates: age > 63 years, male sex, cardiac disease, diabetes, hypertension, CDDP dose > 75 mg/m^2^, Mg supplementation, regular use of NSAIDs, ECOG PS, and the first cycle of chemotherapy). The cutoff values for age (63 years) and cisplatin dose (75 mg/m^2^) were those obtained in a previous study^22^. For confirmation, these cutoff values were also calculated for the present study population with similar results. Patients were matched for variables at a 1:1 ratio using a caliper width of 0.2 of the standard deviation from the propensity score logit.

In the matched cohort of 396 patients, we compared the incidence of nephrotoxicity between the two groups using the Chi-square test. Furthermore, we evaluated the rate of CCr or SCr change by comparing whether the two-diuretic group was superior to the one-diuretic group by comparing the indices of nephrotoxicity after CDDP administration.

The rates of CCr and SCr change were calculated using the following formula:

$$ {\text{SCr}}: \, (\left[ {{\text{maximum SCr}}\_{\text{baseline SCr}}} \right]\, \times \,{1}00/{\text{baseline SCr}}) $$

$$ {\text{CCr}}: \, (\left[ {{\text{maximum CCr}}\_{\text{baseline CCr}}} \right]\, \times \,{1}00/{\text{baseline CCr}}) $$

We assessed the independent risk factors for nephrotoxicity using logistic regression analysis to control for the following potential risk factors: age > 63 years, heart disease, hypertension, diabetes, CDDP dose > 75 mg/m^2^, male sex, concomitant NSAIDs, Mg supplementation, and two diuretics. Statistical significance was set at P < 0.05. All statistical analyses were performed using JMP 14.3.0 software (SAS Institute, Cary, NC, USA).

### Research involving human participants

All procedures performed in studies involving human participants were in accordance with the ethical standards of the institutional research committees and with the 1964 Declaration of Helsinki and its later amendments or comparable ethical standards.

### Consent to participate

The requirement for informed consent was waived due to the retrospective nature of the study.

## Results

### Patient characteristics

**Figure 1:**
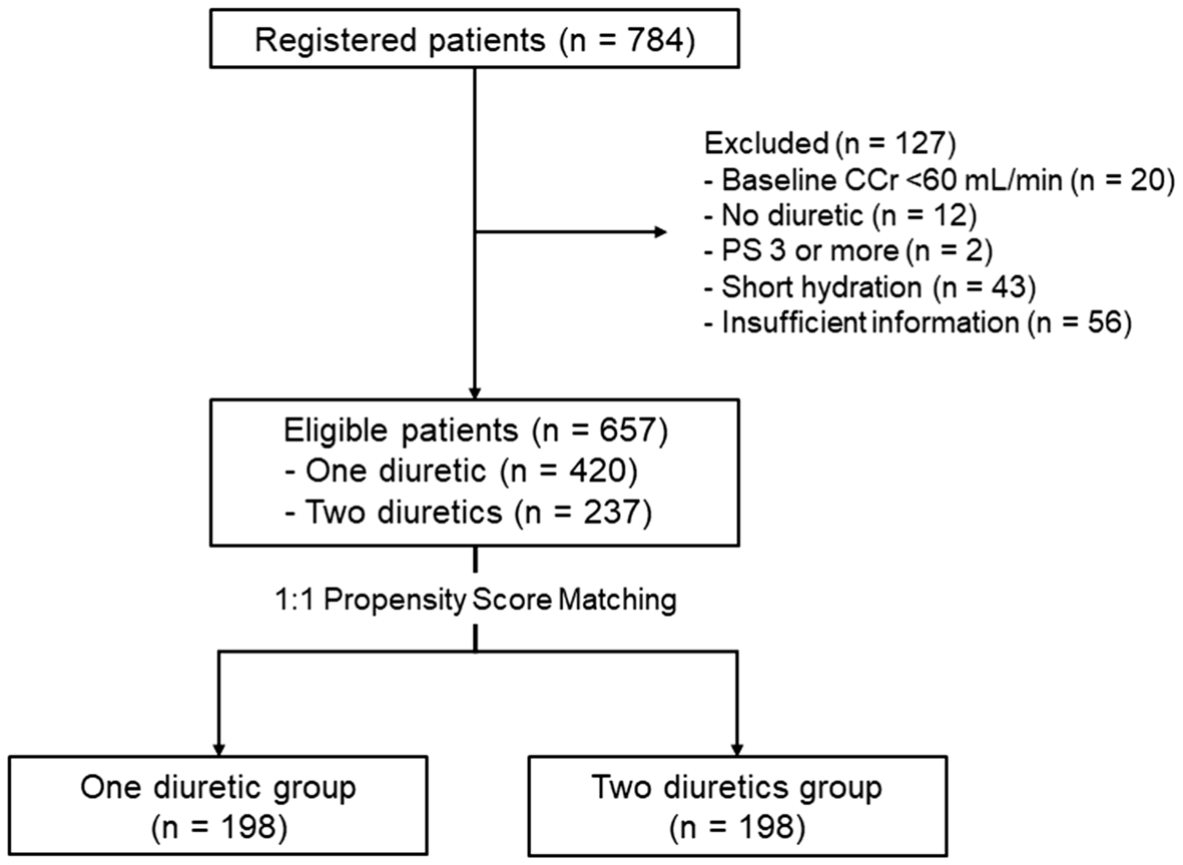
Patient enrollment flowchart. *CCr* creatinine clearance, *PS* performance status.

We analyzed the data of 396 matched patients: 198 received one diuretic (furosemide or mannitol) and 198 received two diuretics (furosemide and mannitol) (Fig. 1). The patient backgrounds before and after adjustment for propensity score matching are shown in Table 1. There were no significant differences in these characteristics between the two groups after propensity score matching. There was no difference in baseline CCr (mL/min) values between the two groups (85.4 ± 16.1 vs. 84.7 ± 17.3, P = 0.507).

**Table 1:** Patient characteristics.

	Before matching					After matching				
	One diuretic		Two diuretics		P	One diuretic		Two diuretics		P
	n = 420		n = 237			n = 198		n = 198		
	n	%	n	%		n	%	n	%	
Sex										
Male	339	80.7	166	70.0	0.002	145	73.2	146	73.7	0.909
Female	81	19.3	71	30.0		53	26.8	52	26.3	
Age (years)										
≥ 63	203	48.3	145	61.2	0.002	107	54.0	113	57.1	0.544
< 63	217	51.7	92	38.8		91	46.0	85	42.9	
CDDP dose										
≥ 75 mg/m^2^	274	65.2	94	39.7	< 0.001	93	47.0	91	46.0	0.840
< 75 mg/m^2^	146	34.8	143	60.3		105	53.0	107	54.0	
Cardiac disease										
Yes	26	6.2	16	6.8	0.868	11	5.6	14	7.1	0.535
No	394	93.8	221	93.2		187	94.4	184	92.9	
Diabetes										
Yes	43	10.2	29	12.2	0.438	18	9.1	19	9.6	0.863
No	377	89.8	208	87.8		180	90.9	189	90.4	
Hypertension										
Yes	107	25.5	64	27.0	0.711	58	29.3	54	27.3	0.655
No	313	74.5	173	73.0		140	70.7	144	72.7	
Mg supplementation										
Yes	216	51.4	133	56.1	0.256	113	57.1	110	55.6	0.761
No	204	48.6	104	43.9		85	42.9	88	44.4	
NSAIDs (regular use)										
Yes	89	21.2	56	23.6	0.494	40	20.2	49	24.8	0.279
No	331	78.8	181	76.4		158	79.8	149	75.2	
ECOG PS										
0	313	74.5	116	49.0	< 0.001	112	56.6	109	55.1	0.762
≥ 1	107	25.5	121	51.0		86	43.4	89	44.9	
Number of cycles										
1	91	21.7	23	9.7	< 0.001	15	7.6	21	10.6	0.294
≥ 2	329	78.3	214	90.3		183	92.4	177	89.4	

*CDDP* cisplatin, *ECOG PS* Eastern Cooperative Oncology Group performance status, *NSAID* nonsteroidal anti-inflammatory drugs, *Mg* magnesium.

### Incidence of nephrotoxicity

The incidence of nephrotoxicity in each group after adjustment is presented in Table 2. There were no significant differences according to CTCAE ver. 5.0 grading between the two groups (P = 0.416).

**Table 2:** Severity of nephrotoxicity.

	One diuretic		Two diuretics		P
	n = 198		n = 198		
	n	%	n	%	
Grade 0	154	77.8	142	71.7	0.416
Grade 1	38	19.2	46	23.2	
Grade 2	6	3.0	9	4.6	
Grade 3	0	0	1	0.5	

### Changes in SCr and CCr in all subsequent cycles

**Figure 2:**
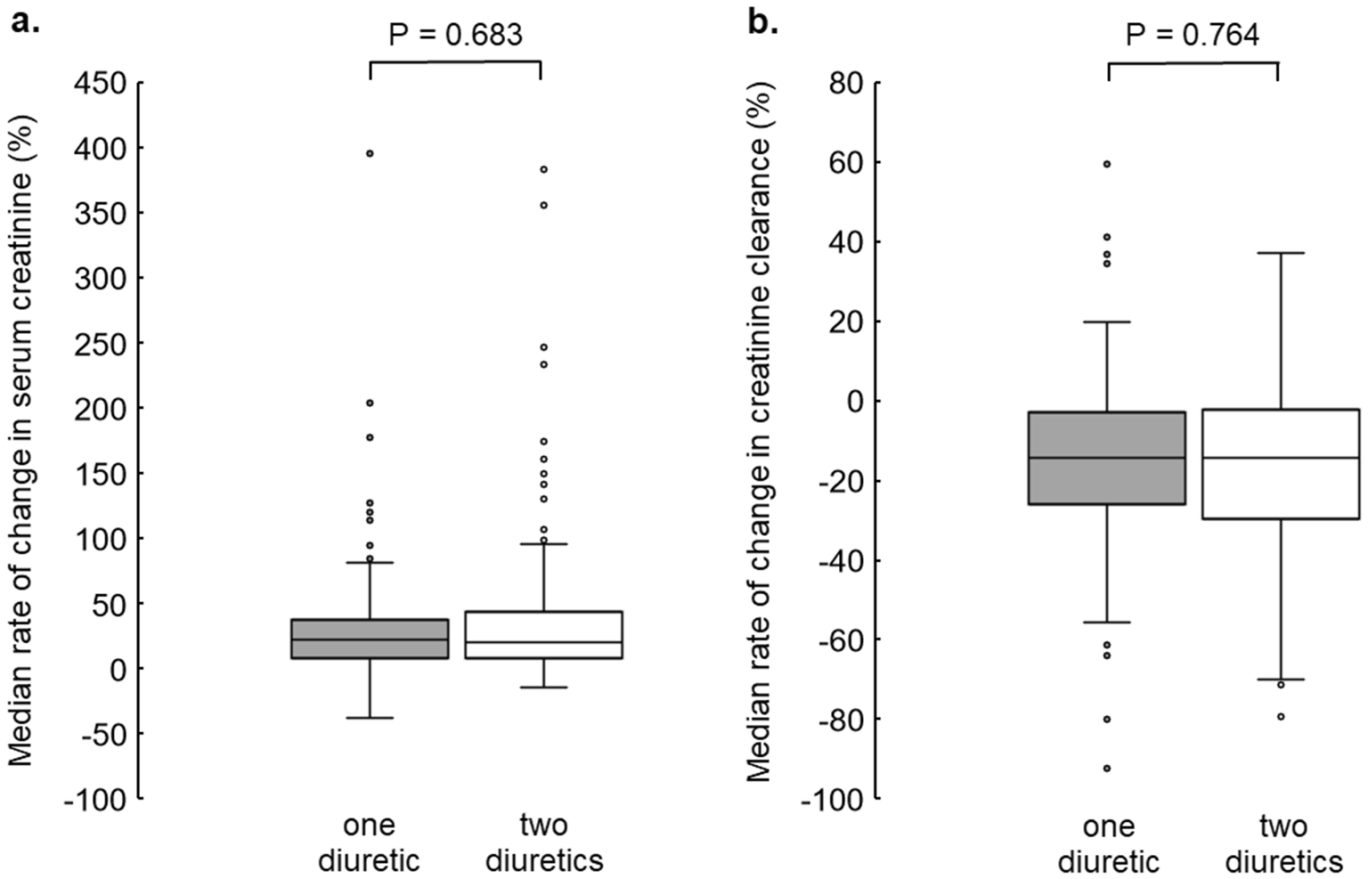
Comparison of the rate of change in SCr and CCr between the one- and two-diuretic groups in all subsequent cycles. Box-and-whisker plots show the relationship between one- and two-diuretic groups and the median rate of change in SCr (**a**) and CCr (**b**) during subsequent cycles of CDDP-based chemotherapy. Differences between the two groups were analyzed using the Mann–Whitney U test. *SCr* serum creatinine level, *CCr* creatinine clearance, *CDDP* cisplatin.

There were no significant differences in the rates of SCr and CCr change between the two groups in all subsequent cycles (P = 0.683 and P = 0.764, respectively) (Fig. 2).

### Risk factors for nephrotoxicity

The results of univariate and multivariate logistic regression analyses of the risk factors for nephrotoxicity are shown in Table 3. Hypertension (P = 0.003), CDDP dose ≥ 75 mg/m^2^ (P = 0.018), and no Mg supplementation (P = 0.002) were identified as independent risk factors for CDDP-induced nephrotoxicity.

**Table 3:** Risk factors for CDDP-induced nephrotoxicity.

	Univariate			Multivariate		
	OR	95% CI	P	OR	95% CI	P
Sex						
Male vs. female	1.283	0.754–2.181	0.357	1.280	0.728–2.250	0.391
Age (years)						
≥ 63 vs. < 63	0.970	0.615–1.531	0.897	0.754	0.461–1.233	0.260
CDDP dose (mg/m^2^)						
≥ 75 vs. < 75	1.420	0.901–2.238	0.130	1.885	1.113–3.194	0.018
Cardiac disease						
Yes vs. no	1.731	0.740–4.050	0.201	1.641	0.666–4.043	0.282
Diabetes						
Yes vs. no	0.947	0.431–2.081	0.891	0.791	0.339–1.843	0.586
Hypertension						
Yes vs. no	2.074	1.283–3.352	0.003	2.173	1.298–3.638	0.003
Mg supplementation						
Yes vs. no	0.638	0.405–1.007	0.052	0.424	0.249–0.725	0.002
NSAIDs (regular use)						
Yes vs. no	1.122	0.657–1.916	0.673	1.288	0.731–2.268	0.381
Forced diuresis						
Two diuretics vs. one diuretic	1.380	0.875–2.178	0.165	1.412	0.881–2.263	0.152

*CI* confidence interval, *OR* odds ratio, *CDDP* cisplatin, *NSAID* nonsteroidal anti-inflammatory drugs, *Mg* magnesium.

## Discussion

In this study, we compared the efficacy of two diuretics (furosemide and mannitol) versus one diuretic alone (furosemide or mannitol) in preventing CDDP-induced nephrotoxicity. There were no significant differences in the incidence of nephrotoxicity or changes in SCr or CCr levels between the two groups. Hypertension, CDDP dose ≥ 75 mg/m^2^, and no Mg supplementation were identified as risk factors for nephrotoxicity, whereas the number of diuretics was not.

CDDP-induced nephrotoxicity is a dose-dependent toxicity, and in this study, CDDP dose (≥ 75 mg/m^2^) was a risk factor for nephrotoxicity, consistent with the results of previous reports^23,24^. Regarding hypertension, chronic systemic hypertension accelerates renal aging^25^. Furthermore, renal atherosclerosis is more common in patients with hypertension, and hypertensive nephrosclerosis is associated with chronic ischemic damage to the tubulointerstitium, a major site of CDDP-induced nephrotoxicity^26,27^. These results suggest that the nephrotoxicity due to high-dose CDDP is exacerbated in patients with hypertension, and antihypertensive drugs may also affect nephrotoxicity in patients with a history of hypertension. Regarding Mg supplementation, hypomagnesemia is a well-known side effect of CDDP-based chemotherapy. Several studies have reported that Mg supplementation reduces CDDP-induced nephrotoxicity by preventing hypomagnesemia^6–8,10,11^. The results of the present study were consistent with those of previous reports. In addition, Mg supplementation may be more important in patients with hypertension because hypertension is reported to be a risk factor for hypomagnesemia^5^.

In this study, the combination of two diuretics did not reduce nephrotoxicity compared with a single diuretic, indicating that the number of diuretics plays a less important role in renal protection against CDDP-induced toxicity than other interventions do, such as hydration or Mg supplementation. In fact, the concomitant use of two diuretics may have resulted in excessive water excretion, leading to increased plasma concentrations of CDDP that may have offset the preventive effect of hydration on nephrotoxicity. Furosemide and mannitol have been reported to prevent nephrotoxicity in vivo; however, the evidence in humans remains unclear. Although there is insufficient robust evidence regarding the efficacy of diuretics in preventing CDDP-induced nephrotoxicity, diuretics are administered with every course of CDDP unless serious side effects or allergic reactions to diuretics develop in clinical practice. If diuretics cannot be administered, a possible approach is to monitor the patient's urine output and adjust the amount of hydration and/or Mg administration, but situations in which none of the diuretics can be used are considered rare. The role of diuretics among various preventive methods for CDDP-induced nephrotoxicity, such as hydration and Mg administration, is unclear. Further research is needed on the role of diuretics in the prevention of CDDP-induced nephrotoxicity. Considering the effect of diuretics in preventing kidney damage, the side effects of diuretics, and the risk of polypharmacy, we could not find a benefit of the use of two diuretics.

This study had several limitations. First, this was a retrospective observational study rather than a randomized or prospective study. Second, individual quantifiable data on heart disease (e.g., cardiac output and ejection fraction) were not available; therefore, we defined heart disease only based on a history of heart disease, such as angina or myocardial infarction. Third, data on serum Mg levels, blood glucose and blood pressure, urine dipsticks for hematuria or proteinuria, and urine volume were not available, and it was not possible to adjust for the time of blood creatinine measurement because of the observational nature of the study. Fourth, data on potential risk factors such as use of H2-receptor inhibitors, metformin, contrast agents, angiotensin-converting enzyme inhibitors, and angiotensin II receptor blockers were not available. Fifth, the safety profile could not be determined in this pooled analysis because data on adverse events were not available in the medical records. Sixth, clinical testing was performed in all cases immediately before each cycle of chemotherapy, whereas testing during the treatment cycle varied from case to case. As a result, the KDIGO criteria for AKI could not be used to assess nephrotoxicity in this study. Although we recognize the importance of the KDIGO criteria in assessing the details of the development of renal injury, the CTCAE is a standard measure of chemotherapy-induced toxicity in clinical oncology, and we consider it has some relevance in this study. Finally, the inclusion of only conventional high-volume hydration and not the short hydration method as a method for preventing nephrotoxicity other than forced diuresis limits the generalizability of the study results.

In conclusion, we did not find any advantage in combining furosemide and mannitol over the use of a single diuretic (furosemide or mannitol) in preventing CDDP-induced nephrotoxicity. Patients receiving high-dose CDDP-based chemotherapy (≥ 75 mg/m^2^) or those with hypertension should be monitored carefully for renal function, and Mg supplementation should be prescribed. Further randomized trials are needed to determine the optimal use of diuretics to prevent CDDP-induced nephrotoxicity.

### Supplementary Information

Supplementary Tables.

## Data Availability

Data supporting the findings of this study are available from the corresponding author upon request. However, restrictions apply to the availability of these data, as they were used under a license for the current study and are thus not publicly available.
