# Supplementary material for: Comparison of preventive effects of combined furosemide and mannitol versus single diuretics, furosemide or mannitol, on cisplatin-induced nephrotoxicity
Source: Sci Rep. 2024 May 7;14:10511. doi: 10.1038/s41598-024-61245-6 (PMC11076568; doi:10.1038/s41598-024-61245-6)
Supplement: Supplementary file 1 — Supplementary Tables. [file 41598_2024_61245_MOESM1_ESM.docx]

Supplement Table 1.

| Cancer type | one diuretic | |  | two diuretics | | Total |
| --- | --- | --- | --- | --- | --- | --- |
|  | n | % |  | n | % | n |
| Lung | 161 | 38.3 |  | 85 | 35.9 | 246 |
| Head and neck | 135 | 32.1 |  | 8 | 3.4 | 143 |
| Esophageal | 81 | 19.3 |  | 25 | 10.5 | 106 |
| Gastric | 10 | 2.4 |  | 62 | 26.2 | 72 |
| Urothelial | 9 | 2.1 |  | 26 | 11 | 35 |
| Neuroendocrine | 9 | 2.1 |  | 6 | 2.5 | 15 |
| Unkown | 6 | 1.4 |  | 6 | 2.5 | 12 |
| Malignant pleural mesothelioma | 5 | 1.2 |  | 4 | 1.7 | 9 |
| Ovarian | 0 | 0 |  | 7 | 3 | 7 |
| Cervical | 0 | 0 |  | 3 | 1.3 | 3 |
| Osteosarcoma | 3 | 0.7 |  | 0 | 0 | 3 |
| Endometrial | 0 | 0 |  | 2 | 0.8 | 2 |
| Peritoneal | 0 | 0 |  | 2 | 0.8 | 2 |
| Pancreatic | 1 | 0.2 |  | 0 | 0 | 1 |
| Vaginal | 0 | 0 |  | 1 | 0.4 | 1 |

Supplement Table 2.

|  | one diuretic | |  | two diuretics | | Total |
| --- | --- | --- | --- | --- | --- | --- |
|  | n | % |  | n | % | n |
| Chemotherapy regimen |  |  |  |  |  |  |
| CDDP | 107 | 25.5 |  | 6 | 2.5 | 113 |
| CDDP+S-1 | 41 | 9.8 |  | 59 | 24.9 | 100 |
| CDDP+5-FU | 78 | 18.6 |  | 21 | 8.9 | 99 |
| CDDP+CPT-11 | 26 | 6.2 |  | 40 | 16.9 | 66 |
| CDDP+ETP | 46 | 11 |  | 15 | 6.3 | 61 |
| CDDP+PEM | 34 | 8.1 |  | 18 | 7.6 | 52 |
| CDDP+GEM | 12 | 2.9 |  | 26 | 11 | 38 |
| CDDP+VNR | 20 | 4.8 |  | 15 | 6.3 | 35 |
| CDDP+PEM+Bev | 17 | 4 |  | 12 | 5.1 | 29 |
| CDDP+DTX+5-FU | 18 | 4.3 |  | 0 | 0 | 18 |
| CDDP+5-FU+Cmab | 13 | 3.1 |  | 4 | 1.7 | 17 |
| CDDP+CAPE+HER | 1 | 0.2 |  | 13 | 5.5 | 14 |
| CDDP+CAPE | 1 | 0.2 |  | 3 | 1.3 | 4 |
| CDDP+DTX | 0 | 0 |  | 3 | 1.3 | 3 |
| CDDP+DXR | 3 | 0.7 |  | 0 | 0 | 3 |
| CDDP+S-1+HER | 0 | 0 |  | 2 | 0.8 | 2 |
| CDDP+AMR | 1 | 0.2 |  | 0 | 0 | 1 |
| CDDP+GEM+BV | 1 | 0.2 |  | 0 | 0 | 1 |
| CDDP+MTX+DXR+VLB | 1 | 0.2 |  | 0 | 0 | 1 |
| Diuretics |  |  |  |  |  |  |
| Furosemide+Mannitol | － | － |  | 237 | 100 | 237 |
| Furosemide | 225 | 53.6 |  | － | － | 225 |
| Mannitol | 195 | 46.4 |  | － | － | 195 |

5-FU, Fluorouracil; AMR, Amrubicin; BEV, Bevacizumab; BLM, Bleomycin; CAPE, Capecitabine; Cmab, Cetuximab; CPT-11, Irinotecan, DTX, Docetaxel; DXR; Doxorubicin, ETP, Etoposide;

GEM, Gemcitabine; HER, Trastuzumab; MTX, Methotrexate; PEM, Pemetrexed;

S-1, Tegafur+Gimeracil+Oteracil Potassium; VLB, Vinblastine; VNR, Vinorelbine.
